# Adolescent leisure reading and its longitudinal association with prosocial behavior and social adjustment

**DOI:** 10.1038/s41598-023-35346-7

**Published:** 2023-06-15

**Authors:** Jan Lenhart, Tobias Richter, Markus Appel, Raymond A. Mar

**Affiliations:** 1 Department of Psychology, University of Bamberg, Bamberg, Germany; 2 Department of Psychology IV, University of Würzburg, Würzburg, Germany; 3 Department of Human-Computer-Media, University of Würzburg, Würzburg, Germany; 4 Department of Psychology, York University, Toronto, Canada

**Keywords:** Human behaviour, Psychology

## Abstract

**Abstract:**

Reading is a popular leisure activity for children, teenagers, and adults. Several theories agree that reading might improve social cognition, but the empirical evidence remains tentative, with research on adolescents especially lacking. We employed a very large, and nationally representative, longitudinal dataset from the National Educational Panel Study (NEPS) in Germany to examine this hypothesis. Specifically, we tested whether reading prospectively predicted future self-reported prosocial behavior and social adjustment in adolescents, controlling for a number of covariates. Two-way cross-lagged panel analyses probed the longitudinal relationship between leisure reading and these social outcomes from Grade 6 to Grade 9. In addition, we examined the effect of cumulative reading experience across Grades 5–8 on future social outcomes, using structural equation modeling. We also explored the unique contributions of cumulative reading experience in different literary genres (classic literature, popular literature, nonfiction, comic books). Cumulative reading in general did not predict future prosocial behavior and social adjustment. However, cumulative reading of modern classic literature was positively associated with later prosocial behavior and social adjustment.

**Protocol registration:**

The stage 1 protocol for this Registered Report was accepted in principle on 08 November 2021. The protocol, as accepted by the journal, can be found at: 10.17605/OSF.IO/KSWY7.

## Introduction

From birth onwards, children are virtually engulfed in stories, with parents and grandparents engaging them in shared reading. Later, when children can read on their own, they become more and more independent, choosing their own reading material and developing their own reading habits. In Germany, these habits involve frequent leisure reading, with around one third of twelve- to nineteen-year-olds reading for fun several times a week, or even daily^1^. Although research has repeatedly demonstrated the benefits of reading in terms of verbal ability and academic achievement^2^, reading might also benefit social-cognitive development.

The idea that stories might improve social abilities is ancient and can be traced back to the invention of written fiction^3,4^. In characterizing these historical ideas, Hakemulder speaks of stories as a kind of moral laboratory, in which readers exercise their social abilities and consequently become better human beings^5^. Following this idea, many current theories agree that stories could serve to improve social-cognitive skills and relevant behaviors, such as theory-of-mind, empathy, and prosocial behavior^6–10^.

However, the state of the empirical evidence supporting this idea remains tentative, with most studies focusing solely on theory-of-mind or empathy and predominantly relying upon cross-sectional correlations^11^ or single-session experiments of short-term effects^12^. The main goal of the present study is to use longitudinal data to examine the relationship between leisure reading and real-life consequences of social cognition, namely prosocial behavior and social adjustment. In addition to being longitudinal, this dataset is also based on a large, nationally-representative, sample from Germany. Moreover, past studies almost exclusively study young adult student populations or, to a lesser degree, children. This neglects the pivotal period of adolescence, when crucial social-cognitive development takes place^13^. Thus, our second goal is to focus on the relationship between reading and social outcomes during adolescence, with our sample comprised of grade-school children (followed from around age 10 to around age 15). Finally, although current theories agree that stories might improve social cognition, they disagree whether all forms of narrative literature or only “literary” texts might have these effects^6,8^. Thus, our final goal is to explore any differential effects related to genre.

In the following, we first provide a short overview of theories for why reading might aid the development of social-cognitive skills and related behavior. We then discuss the available empirical evidence for this idea, as well as why different genres might result in different effects.

Most relevant theories agree that engaging with stories that comprise social content might improve social cognition and related behavior^7,8^. For example, the Social Processes and Content Entrained by Narrative (SPaCEN) framework emphasizes the role of narrativity and proposes two ways in which narratives could promote social cognition^8^: (1) through honing social processes and/or (2) presenting social content. With respect to social processes, stories contain characters, which act as targets that provide opportunities to practice and hone social-cognitive processes. Based on Oatley’s conception of stories as simulations of other minds^9^, the SPaCEN framework theorizes that frequently inferring characters’ mental states might improve the ability to do the same with real-world peers^8^. An alternative, but not mutually exclusive, path for stories to improve social cognition is by communicating concrete social knowledge. The SPaCEN framework proposes that stories contain social information—such as knowledge about complex emotions like grief—and this information could be learned and then applied in the real world. This is in line with Bandura’s social cognitive theory, with stories modelling real-world situations, allowing readers to learn about appropriate social behavior^14,15^. Whereas the process account describes how social cognitive functions could be bolstered or improved, the content account describes how the information upon which these functions operate could be learned from stories. Both the process and the content account predict that stories could improve social cognition, and by doing so might also lead to prosocial behavior and better social adjustment.

Prosociality and social adjustment are potential downstream consequences of social cognition, in that those with better social cognition have the potential be more considerate of others and use their knowledge of others to better fit in. Empirical research has largely confirmed that social cognition predicts these outcomes. For example, a meta-analysis of 76 studies of children (aged 2–12) confirmed that better theory-of-mind abilities predict prosocial behavior^16^. Related work also ties social-cognitive ability to better social adjustment. Those higher in empathy are less likely to be bullies (although theory-of-mind is unassociated)^17^, those higher in theory-of-mind at age 5 are less likely to be friendless at age 7^18^, and theory-of-mind is also associated with popularity (among children aged 2–10)^19^. Based on these associations in childhood, it seems reasonable to expect that if stories promote social cognition in adolescence, we should also observe benefits for prosociality and social adjustment. However, as these are somewhat distal outcomes, the SPaCEN framework notes that associations with story exposure should be weaker than what is observed for direct measures of social cognition (p. 461)^8^. Weaker associations are also expected given the many other contributors to these outcomes and various complicating factors at play (e.g., motivation).

Although the SPaCEN framework argues that the inclusion of social content within a narrative structure is all that is required for stories to promote social cognition^8^, others disagree. Some theories argue that it is the literary nature of the texts that is most relevant for fostering social-cognitive development^6^. Theoretically, literariness can be defined in a number of different ways, such as higher levels of phonetic, grammatical, or semantic foregrounding leading to greater defamiliarization^20,21^; a polyphony of perspectives^22^; or gaps that need to be filled by “a search for meanings among a spectrum of possible meanings” (p. 25)^23^. The common core of these perspectives is that literariness is assumed to promote conscious, effortful processing of the text and deeper reflections on the content. For theorists like Kidd and Castano, this is what is necessary to foster social-cognitive skills and behavior^6^.

Most of the empirical evidence concerning whether stories promote social-cognition stems from cross-sectional correlational data^24–27^ or experiments examining short-term effects^6,28,29^. A meta-analysis of the correlational studies by Mumper and Gerrig found that reading fiction predicts self-reported empathy (*r* = 0.07), fantasy (i.e., the degree to which a reader becomes immersed in stories; *r* = 0.18), and performance on theory-of-mind tasks (*r* = 0.21)^11^. For reading nonfiction, the correlations are weaker, but also statistically significant (empathy, *r* = 0.05; fantasy, *r* = 0.05; theory-of-mind, *r* = 0.09). Thus, correlational studies find small to moderate relationships between leisure reading and various aspects of social cognition, with these associations higher for fiction than for nonfiction. However, correlations do not allow for conclusions regarding the causal direction of effects and cannot rule out third-variable explanations. For instance, fiction and nonfiction reading habits tend to be highly correlated^26,30^, with frequent fiction readers also consuming more nonfiction (and vice versa). Unfortunately, Mumper and Gerrig’s meta-analysis could not take this shared variance into account, or any other potential confounding variable, due to the primary studies available^11^. As a result, it is not known if the associations between nonfiction and social outcomes are a function of fiction, or if the relationships between fiction and these same outcomes are explained by other factors.

Only a few studies have attempted to account for potential third-variable explanations. In these studies, after controlling for nonfiction, fiction exposure is associated with better performance on theory-of-mind and empathy tasks^26^, and predicts self-reported cognitive empathy^30^. In contrast, after controlling for fiction, nonfiction shows null or even negative correlations with these same measures^26,30^. In addition, the relationship between reading fiction and social-cognitive skills remains statistically significant when a number of individual differences are statistically controlled (e.g., gender, age, Openness to Experience, intelligence^24,26,31^; but see also contradictory findings^25^).

The other predominant way in which this topic has been studied is with single-session experiments. In these experiments, participants typically read one short story or an excerpt from a book and then directly complete one or more social-cognitive tasks^6,28,29,32,33^. Unlike cross-sectional correlational studies, these experiments can support causal inferences and rule out potential third variable explanations, when properly conducted. A meta-analysis of these experiments found that fiction reading improves social-cognitive performance relative to nonfiction reading or no reading (*g* = 0.15)^12^. However, the findings from these experiments suffer from several major shortcomings. First, most of these experiments do not assess the durability of these effects, looking only at immediate outcomes in the short-term. Thus, it is not clear whether reading narratives lead to improvements that represent meaningful long-term gains in social-cognitive abilities, or just temporary changes that could reflect a kind of priming or social cueing effect. Second, reading a single short story or an excerpt^6,28,29,32,33^ or even an entire book^34^, does not reflect the theoretical accounts of these phenomena^8,9^. Theoretically, the social effects of reading are assumed to emerge from cumulative, repeated exposure to stories over long periods of time^6,8^.

Longitudinal research designs do a far better job of examining cumulative effects like those proposed for reading narrative fiction. Unfortunately, to our knowledge, at the time of this registered report there exist only a handful of longitudinal studies that have looked at this question. Van Schooten and de Glopper investigated the development of literary response (i.e., cognitive responses to texts by readers) in Dutch secondary school students (Grades 7 to 11)^35^. They found that the amount of leisure reading predicted changes in self-reported empathy for fictional characters, from Grade 8 to 9, Grade 10 to 11, and from Grade 9 to 11 (but not from Grade 7 to 8, or Grade 7 to 9). Although certainly encouraging, this study relies entirely on a self-report measure of empathy for fictional characters, and does not examine any real-world social cognition or related behavior. Nor did this study control for any potential third-variable explanations. A second longitudinal study on this topic used a propensity score matching analysis to analyze data from the UK Millennium Cohort Study^36^. These researchers found that reading frequency at the age of 7 predicted children’s prosocial behavior at age 11. Importantly, potential third variable explanations were controlled by matching participants on a number of individual, social, familial, and behavioral characteristics. However, they did not find that reading predicted other related outcomes, such as fewer emotional problems, less difficulty with peers, or fewer issues related to conduct. These null findings indicate that either the influence of leisure reading does not extend to these outcomes, or that associations with these outcomes are weak and could not be detected in this study. In addition, this analysis did not examine the potential for differential effects based on genre, such as comparing popular and literary fiction to nonfiction, as in our study.

In sum, prior evidence from cross-sectional correlational, and experimental studies suffer from several limitations, and together do not adequately answer whether reading fosters pro-social behavior and social adjustment in the long run. Long-term longitudinal studies that span many years are a better fit for this research question, and the results from prior studies of this type are certainly promising. Our proposed study improves upon and expands the purview of this past work by studying adolescents, controlling for third-variable explanations, and examining possible differential effects based on genre.

Although the SPaCEN framework proposes that all narratives have equal potential to foster social cognition, other theorists propose that this potential is limited to literary fiction^6^. Empirically, however, evidence regarding the importance of literariness is decidedly mixed. In terms of correlational studies, one examination looked at lifetime exposure to different literary genres and whether this exposure predicts mental-inferencing ability^24^. Importantly, these researchers attempted to rule out third variable explanations by controlling for trait personality, gender, age, English fluency, and exposure to nonfiction. After accounting for these variables, and in direct contrast to the proposed importance of literariness, only two forms of popular fiction remained predictors: romance and suspense/thriller. However, Kidd and Castano followed a parallel approach in several distinct samples and found just the opposite: after controlling for a number of covariates (e.g., gender, age) only exposure to literary texts, and not popular fiction, predicted social-cognitive performance^32,37^.

With respect to experiments, Kidd and Castano contrasted literary and popular fiction in several studies^6^. Across their experiments, they found that reading literary fiction resulted in better social-cognitive performance than reading popular fiction (Experiments 2–5) and a no-reading control group (Experiments 2 and 5), whereas popular fiction did not differ from the latter (Experiments 2 and 5). Although these findings have been directly and conceptually replicated^29,38^, there are just as many failures to replicate^28,33^, with a recent replication attempt by the same group reporting mixed findings^32^.

In sum, evidence for the idea that literariness might be crucial for the beneficial effects of narrative literature is inconsistent. These studies also share the limitations previously discussed, as they also rely on correlational and single-session experimental designs. Cross-sectional correlation studies cannot evaluate causal direction and are vulnerable to alternative, third-variable explanations. And single-session experimental examinations of short-term effects do not match the theoretical assumption that frequent and prolonged engagement with stories is important in order to foster social benefits^8^. Both shortcomings underline the need for more long-term longitudinal research on this topic.

The goal of the present study is to extend past research by examining potential longitudinal effects of reading on social-cognitive skills and related behavior, in adolescents and in terms of genre, after controlling for key alternative explanations. To do so, we analyzed German data, from the National Educational Panel Study (NEPS). This study collects longitudinal data on educational processes and competencies based on large, nationally-representative, samples. More precisely, we used data from the starting cohort in Grade 5, which follows students all the way to Grade 9. The NEPS provides data on students’ leisure reading habits as well as their prosocial behavior and social adjustment in Grades 6 and 9 (self-reports and parent reports). The structure of this dataset allowed us to examine bidirectional relationships between leisure-reading, prosocial behavior, and social adjustment over time, while also controlling for previous levels of prosociality and adjustment. In line with previous research, we made the following predictions (see Table 1):

1. Students’ leisure reading at Grade 6 will predict future prosocial behavior/social adjustment (in Grade 9), even after controlling for earlier prosocial behavior/social adjustment.
2. Leisure reading for a past time period (Grades 5–8) will cumulatively predict future prosocial behavior/social adjustment (Grade 9), even after controlling for earlier prosocial behavior/social adjustment.

**Table 1:** Design table.

Question	Hypothesis	Sampling plan	Analysis plan	Interpretation given to different outcomes
Does leisure reading predict future prosocial behavior?	Leisure reading predicts future prosocial behavior	Secondary analysis of the NEPS data	Two-way cross-lagged panel design analyses that examine associations between leisure reading (Grade 6 and 9) and prosocial behavior (self-reports; Grade 6 and 9), controlling for non-verbal intelligence, socioeconomic status, and migration background	Models with adequate fit, demonstrating statistically significant positive associations between prior leisure reading and later prosocial behavior would be consistent with the hypothesis. An ill-fitting model, or null or negative associations between these variables would be inconsistent with the hypothesis.
Does leisure reading predict future social adjustment?	Leisure reading predicts future social adjustment	Secondary analysis of the NEPS data	Two-way cross-lagged panel design analyses that examine associations between leisure reading (Grade 6 and 9) and social adjustment (self-reports; Grade 6 and 9), controlling for non-verbal intelligence, socioeconomic status, and migration background	Models with adequate fit, demonstrating statistically significant positive associations between prior leisure reading and later social adjustment would be consistent with the hypothesis. An ill-fitting model, or null or negative associations between these variables would be inconsistent with the hypothesis.
Does cumulative reading predict future prosocial behavior?	Cumulative reading predicts future prosocial behavior	Secondary analysis of the NEPS data	Structural equation models examining associations between leisure reading (Grades 5 to 8) and prosocial behavior (self-reports; Grade 9), controlling for earlier prosocial behavior (self-reports; Grade 6), non-verbal intelligence, socioeconomic status, and migration background	Models with adequate fit, demonstrating statistically significant positive associations between leisure reading and prosocial behavior would be consistent with the hypothesis. An ill-fitting model, or the absence of these associations, would be inconsistent with the hypothesis.
Does cumulative reading predict future social adjustment?	Cumulative reading predicts future social adjustment	Secondary analysis of the NEPS data	Structural equation models that examine associations between leisure reading (Grades 5 to 8) and social adjustment (self-reports; Grade 9), controlling for earlier social adjustment (self-report; Grade 6), non-verbal intelligence, socioeconomic status, and migration background	Models with adequate fit, demonstrating statistically significant positive associations between leisure reading and social adjustment would be consistent with the hypothesis. An ill-fitting model, or the absence of these associations, would be inconsistent with the hypothesis.
Does genre differentially relate to future prosocial behavior?	Cumulative reading of narrative material but not of nonnarrative material predicts future prosocial behavior	Secondary analysis of the NEPS data	Structural equation models that examine associations between leisure reading of different genres (Grades 5, 7, and 8) and prosocial behavior (self-reports; Grade 9), controlling for earlier prosocial behavior (self-reports; Grade 6), non-verbal intelligence, socioeconomic status, and migration background	Models with adequate fit, demonstrating statistically significant positive associations between leisure reading of narrative material and prosocial behavior and nonsignificant or negative associations between leisure reading of nonnarrative material and prosocial behavior, after controlling for prior prosocial behavior, would be consistent with the hypothesis. An ill-fitting model, or the absence of any of these associations, would be inconsistent with the hypothesis.
Does genre differentially relate to future social adjustment?	Cumulative reading of narrative material but not of nonnarrative material predicts future social adjustment	Secondary analysis of the NEPS data	Structural equation models that examine associations between leisure reading of different genres (Grades 5, 7, and 8) and social adjustment (self-reports; Grade 9), controlling for earlier social adjustment (self-report; Grade 6), non-verbal intelligence, socioeconomic status, and migration background	Models with adequate fit, demonstrating statistically significant positive associations between leisure reading of narrative material and social adjustment and nonsignificant or negative associations between leisure reading of nonnarrative material and social adjustment, after controlling for prior social adjustment, would be consistent with the hypothesis. An ill-fitting model, or the absence of any of these associations, would be inconsistent with the hypothesis.

Finally, the NEPS also provides information on exposure to different genres (e.g., classic literature, popular literature, comic books, nonfiction), allowing us to examine how genre relates to social behavior over time. Two alternative hypotheses may be derived from the research literature here. According to theories such as the SPaCEN framework^8^, the narrativity of reading materials should drive potential effects on prosocial behavior and social adjustment. Therefore, these theories would predict positive associations with social outcomes for exposure to classical literature, popular literature, and comic books, but not for nonfiction. In contrast, other theories assume that the literariness of reading materials should drive the effects^6,32,37^. These theories predict positive social outcomes for exposure to classical literature, but for not for the other genres. In line with the SPaCEN framework, we made the following prediction (see Table 1):

3. Leisure reading of narrative literature for a past time period (Grades 5, 7, and 8) will cumulatively predict future prosocial behavior/social adjustment (Grade 9), even after controlling for earlier prosocial behavior/social adjustment.

Moreover, we examined whether these relationships remained after controlling for non-verbal intelligence, socioeconomic status, and migration background^25,31^. The latter is important to control for, as children with a migration background may be delayed with respect to German language comprehension, which impacts leisure reading. Moreover, children with a migration background in Germany show higher levels of internalizing problems^39,40^, which could take the form of worse prosocial behavior or social adjustment.

## Results

### Measurement invariance testing of leisure reading, self-reported prosocial behavior, and peer problems

We tested the SDQ scales of Prosocial Behavior and Peer Problems (self-reports) and leisure reading for their measurement invariance between Grades 6 and 9 (Table 2). As imputed data only provide averaged and therefore approximate information on model fit, we used the original non-imputed data for testing longitudinal measurement invariance. For leisure reading, strict measurement invariance was established. For Prosocial Behavior, weak measurement invariance was established. However, an analysis of partial measurement invariance found that strict invariance could be established for three of the five items. For Peer Problems, weak measurement invariance could not be established. An inspection of the items indicated that one item did not show equal factor loadings across time, but for the other four items strict measurement invariance could be established. Accordingly, for Prosocial Behavior and Peer Problems, the models including partial strict measurement invariance were used for further analyses.

**Table 2:** Tests of Measurement Invariance across Measurement Points for Leisure Reading, Prosocial Behavior, and Peer Problems.

Model	χ^2^	*df*	*p*	Δχ^2^	*df*	*p*	RMSEA (90% CI)	SRMR	CFI	TLI	ΔRMSEA < .015	ΔCFI <|-.01|
Leisure reading												
Configural	*NA*											
Weak	20.261	1	< .001				.064 [.041; .089]	.006	.999	.997		
Strong	50.904	6	< .001	33.494	5	< .001	.040 [.030; .050]	.007	.999	.999	yes	yes
Strict	102.514	8	< .001	55.646	2	< .001	.050 [.041; .059]	.010	.997	.998	yes	yes
SDQ prosocial behavior—self-report												
Configural	43.505	29	.041				.010 [.002; .016]	.013	.999	.998		
Weak	86.759	33	< .001	33.608	4	< .001	.018 [.014; .023]	.017	.995	.993	yes	yes
Strong	478.994	37	< .001	470.087	4	< .001	.050 [.046; .054]	.029	.960	.952	no	no
Strong (partial)^a^	126.525	35	< .001	47.246	2	< .001	.023 [.019; .028]	.019	.992	.989	yes	yes
Strict (partial)^b^	160.223	38	< .001	32.624	3	< .001	.026 [.022; .030]	.022	.989	.987	yes	yes
SDQ peer problems—self-report^c^												
Configural	104.544	27	< .001				.024 [.020; .029]	.023	.987	.979		
Weak	242.805	31	< .001	120.575	4	< .001	.038 [.033; .042]	.032	.965	.949	yes	no
Weak (partial)^d^	133.164	30	< .001	26.430	3	< .001	.027 [.022; .031]	.026	.983	.974	yes	yes
Strong (partial)^e^	168.992	33	< .001	32.183	3	< .001	.029 [.025; .034]	.026	.977	.969	yes	yes
Strict (partial)^f^	201.716	37	< .001	34.128	4	< .001	.030 [.026; .034]	.029	.973	.967	yes	yes
SDQ prosocial behavior—parental report												
Configural	73.153	29	< .001				.021 [.015; .027]	.028	.991	.986		
Weak	69.873	33	< .001	2.552	4	.635	.018 [.012; .024]	.028	.992	.989	yes	yes
Strong	76.419	37	< .001	6.458	4	.168	.018 [.012; .023]	.028	.992	.990	yes	yes
Strict	78.836	42	< .001	3.745	5	.587	.016 [.010; .021]	.028	.992	.992	yes	yes
SDQ peer problems—parental report												
Configural	165.104	29	< .001				.037 [.032; .043]	.039	.977	.964		
Weak	275.361	33	< .001	93.541	4	< .001	.046 [.041: .051]	.046	.959	.944	yes	no
Weak (partial)^d^	167.852	32	< .001	7.004	3	.072	.035 [.030; .041]	.039	.977	.968	yes	yes
Strong (partial)^e^	177.477	35	< .001	11.626	3	.009	.034 [.030; .040]	.040	.976	.969	yes	yes
Strict (partial)^f^	171.082	39	< .001	1.177	4	.882	.031 [.027; .036]	.040	.978	.974	yes	yes

Longitudinal measurement invariance testing was conducted with the original non-imputed data. Residual correlations were allowed between identical items across measurement points. To allow for an assessment of weak measurement invariance of leisure reading, the residual correlation between the measurement points for the item on leisure reading during the school days, which was not significant, was set to zero.

^a^Thresholds for items i and g were freely estimated. ^b^Thresholds and residual variances for items i and g were freely estimated. ^c^Residuals of inverted items e and f were allowed to correlate at both measurement points ^d^Factor loading for item h was freely estimated. ^e^Factor loading and threshold for item h were freely estimated. ^f^Factor loading, threshold, and residual variance for item h were freely estimated.

### Cross-lagged relations between leisure reading and self-reported prosocial behavior and peer problems

**Figure 1:**
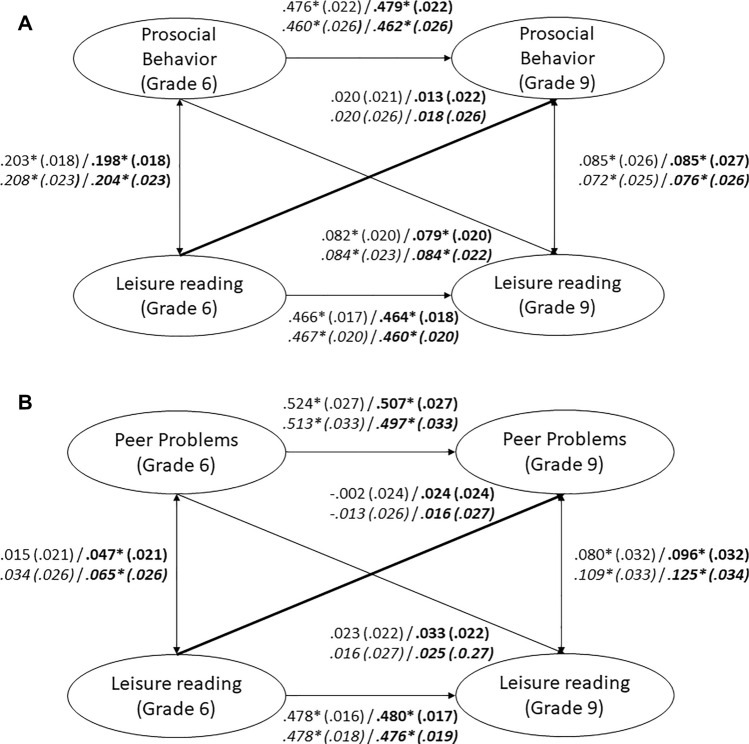
(**A**) Cross-Lagged Panel Model between Leisure Reading and Self-Reported Prosocial Behavior. Numbers represent standardized coefficients with standard errors in parentheses. Coefficients for models that include control variables (migration background, socioeconomic status, nonverbal intelligence) are printed in bold. Coefficients for the reduced sample of students nested in schools are in italics. Model fit: χ^2^ = 409.080, *df* = 82, RMSEA = .028, SRMR = .022, CFI = .994, TLI = .993. Model fit with control variables: χ^2^ = 484.344, *df* = 112, RMSEA = .025, SRMR = .035, CFI = .993, TLI = .992. Nested in schools: Model fit: χ^2^ = 213.987, *df* = 82, RMSEA = .022, SRMR = .022, CFI = .995, TLI = .995. Model fit with control variables: χ^2^ = 277.080, *df* = 112, RMSEA = .021, SRMR = .038, CFI = .994, TLI = .993.* *p* < .05 (two-tailed). (**B**) Cross-Lagged Panel Model between Leisure Reading and Self-Reported Peer Problems. Numbers represent standardized coefficients with standard errors in parentheses. Coefficients for models that include control variables (migration background, socioeconomic status, nonverbal intelligence) are printed in bold. Coefficients for the reduced sample of students nested in schools are in italics. Model fit: χ^2^ = 706.685, *df* = 81, RMSEA = .039, SRMR = .036, CFI = .988, TLI = .987. Model fit with control variables: χ^2^ = 793.656, *df* = 111, RMSEA = .035, SRMR = .043, CFI = .987, TLI = .985. Nested in schools: Model fit: *χ*^2^ = 450.868, *df* = 81, RMSEA = .037, SRMR = .039, CFI = .986, TLI = .984. Model fit with control variables: *χ*^2^ = 515.007, *df* = 111, RMSEA = .033, SRMR = .047, CFI = .984, TLI = .981. * *p* < .05 (two-tailed).

Cross-lagged panel models indicated that leisure reading at Grade 6 and Grade 9 was positively correlated to concurrent self-reported prosocial behavior (Fig. 1A) and peer problems (Fig. 1B). These correlations were small. However, we found no significant effect of leisure reading on later self-reported prosocial behavior (Fig. 1A) or peer problems (Fig. 1B). In addition, we found a significant effect of prosocial behavior on later leisure reading (Fig. 1A). Thus, our hypotheses were not supported. Additional analyses with students nested in schools and including only those students who remained in the same school from Grades 5 to 9 yielded comparable results (see Fig. 1A and B).

### Cumulative leisure reading and self-reported prosocial behavior and peer problems

**Figure 2:**
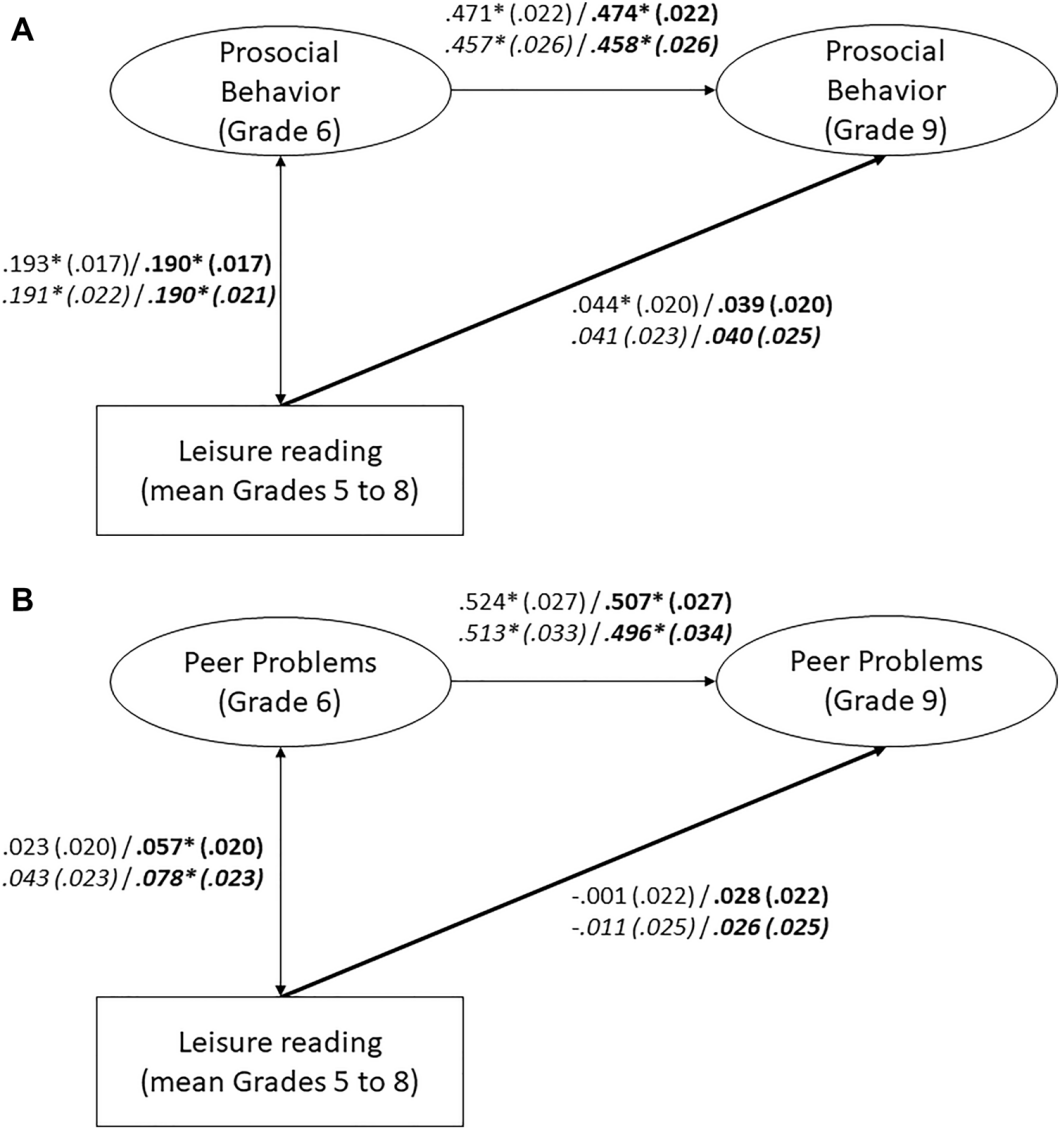
(**A**) Cumulative Leisure Reading and Self-Reported Prosocial Behavior. Numbers represent standardized coefficients with standard errors in parentheses. Coefficients for models that include control variables (migration background, socioeconomic status, nonverbal intelligence) are printed in bold. Coefficients for the reduced sample of students nested in schools are in italics. Model fit: χ^2^ = 267.799, *df* = 46, RMSEA = .031, SRMR = .023, CFI = .985, TLI = .982. Model fit with control variables: χ^2^ = 355.000, *df* = 70, RMSEA = .028, SRMR = .039, CFI = .981, TLI = .976. Nested in schools: Model fit: χ^2^ = 132.619, *df* = 46, RMSEA = .024, SRMR = .021, CFI = .989, TLI = .987. Model fit with control variables: χ^2^ = 194.487, *df* = 70, RMSEA = .023, SRMR = .042, CFI = .984, TLI = .980. * *p* < .05 (two-tailed). (**B**) Cumulative Leisure Reading and Self-Reported Peer Problems. Numbers represent standardized coefficients with standard errors in parentheses. Coefficients for models that include control variables (migration background, socioeconomic status, nonverbal intelligence) are printed in bold. Coefficients for the reduced sample of students nested in schools are in italics. Model fit: χ^2^ = 455.320, *df* = 45, RMSEA = .042, SRMR = .035, CFI = .949, TLI = .937. Model fit with control variables: χ^2^ = 580.232, *df* = 69, RMSEA = .038, SRMR = .045, CFI = .939, TLI = .922. Nested in schools: Model fit: χ^2^ = 271.405, *df* = 45, RMSEA = .039, SRMR = .037, CFI = .946, TLI = .934. Model fit with control variables: χ^2^ = 373.996, *df* = 69, RMSEA = .036, SRMR = .048, CFI = .928, TLI = .908. * *p* < .05 (two-tailed).

Cumulative leisure reading positively predicted later self-reported prosocial behavior when controlling for prior prosocial behavior (Fig. 2A). However, this small effect vanished when the control variables were included. In addition, cumulative leisure reading did not significantly predict later self-reported peer problems (Fig. 2B). Thus, our hypotheses were not supported. Additional analyses with students nested in schools and including only those students who remained in the same school from Grades 5 to 9 yielded comparable results (see Fig. 2A and B).

### Cumulative leisure reading across different genres and self-reported prosocial behavior and peer problems

**Figure 3:**
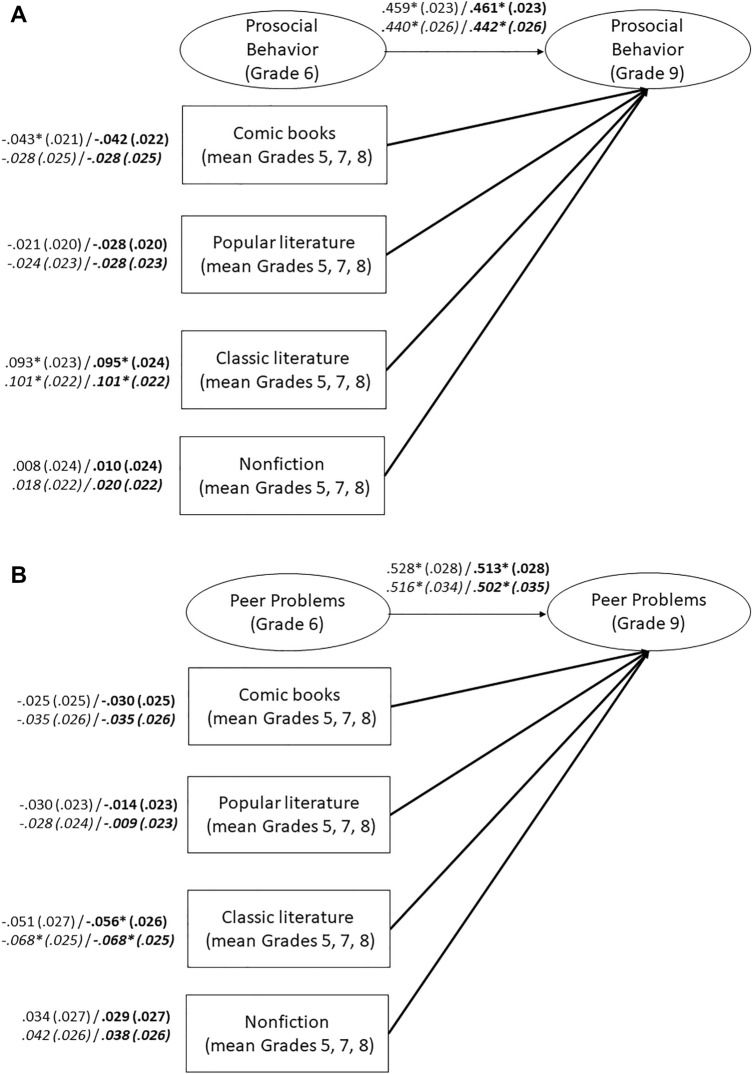
(**A**) Cumulative Leisure Reading across Different Categories and Self-Reported Prosocial Behavior. Numbers represent standardized coefficients with standard errors in parentheses. Coefficients for models that include control variables (migration background, socioeconomic status, nonverbal intelligence) are printed in bold. Coefficients for the reduced sample of students nested in schools are in italics. Model fit: χ^2^ = 416.480, *df* = 70, RMSEA = .031, SRMR = .024, CFI = .978, TLI = .972. Model fit with control variables: χ^2^ = 503.060, *df* = 94, RMSEA = .029, SRMR = .036, CFI = .976, TLI = .966. Nested in schools: Model fit: χ^2^ = 199.042, *df* = 70, RMSEA = .023, SRMR = .022, CFI = .984, TLI = .979. Model fit with control variables: χ^2^ = 268.861, *df* = 94, RMSEA = .024, SRMR = .037, CFI = .979, TLI = .970. * *p* < .05 (two-tailed). (**B**) Cumulative Leisure Reading across Different Categories and Self-Reported Peer Problems. Numbers represent standardized coefficients with standard errors in parentheses. Coefficients for models that include control variables (migration background, socioeconomic status, nonverbal intelligence) are printed in bold. Coefficients for the reduced sample of students nested in schools are in italics. Model fit: χ^2^ = 656.876, *df* = 69, RMSEA = .041, SRMR = .036, CFI = .938, TLI = .919. Model fit with control variables: χ^2^ = 809.302, *df* = 93, RMSEA = .039, SRMR = .042, CFI = .928, TLI = .898. Nested in schools: Model fit: χ^2^ = 327.287, *df* = 69, RMSEA = .033, SRMR = .035, CFI = .943, TLI = .925. Model fit with control variables: χ^2^ = 451.301, *df* = 93, RMSEA = .034, SRMR = .043, CFI = .921, TLI = .887. * *p* < .05 (two-tailed).

Cumulative reading of nonfiction did not predict later self-reported prosocial behavior (Fig. 3A) and peer problems (Fig. 3B). Among the narrative reading categories, only cumulative reading of modern classic literature positively predicted more self-reported prosocial behavior and less self-reported peer problems, when the control variables were included. These effects were small, but statistically significant. However, the similar associations we predicted for popular literature and comic books were not supported. Additional analyses with students nested in schools and including only those students who remained in the same school from Grade 5–9 yielded comparable results (see Fig. 3A and B).

### Exploratory analyses

In exploratory analyses that were not pre-registered, we analyzed the parental reports of Prosocial Behavior and Peer Problems following the same procedure as for the self-reports. In addition, we replicated all analyses with an even more conservative methodological approach, including gender, reading competence, and trait openness to experience as additional control variables.

### Measurement invariance testing of parent reported prosocial behavior and peer problems

We tested the SDQ scales of Prosocial Behavior and Peer Problems (parental reports) for longitudinal measurement invariance between Grades 6 and 9 (Table 2), using the same procedure as for self-reports. For Prosocial Behavior, strict measurement invariance could be established. For Peer Problems, weak measurement invariance could not be established. An inspection of the items indicated that—similar to the self-reports—one item did not show equal factor loadings across time, but for the other four items strict measurement invariance could be established. Accordingly, for Peer Problems, the model including partial strict measurement invariance was used for further analyses.

### Cross-lagged relations between leisure reading and parent reported prosocial behavior and peer problems

**Figure 4:**
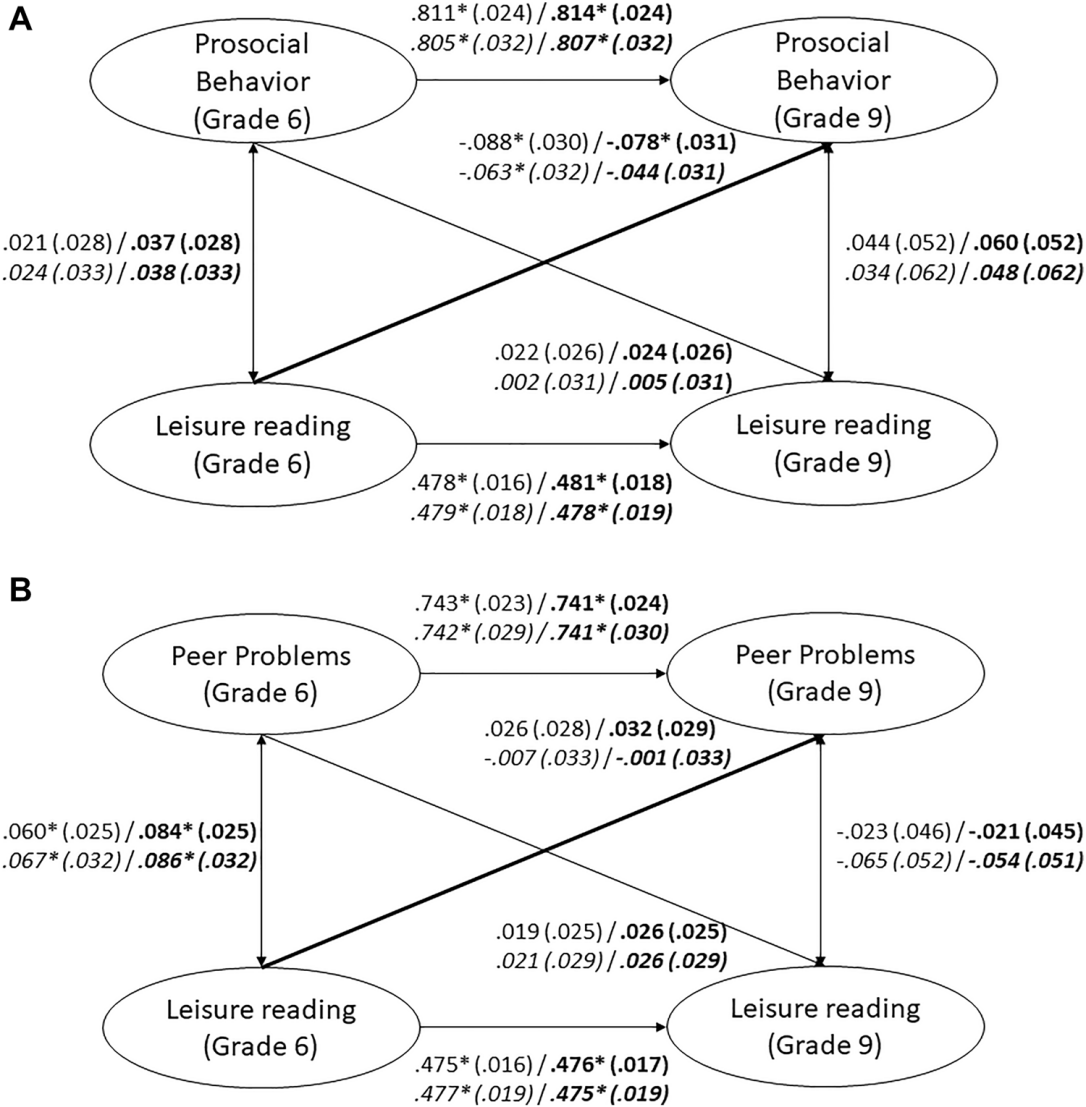
(**A**) Cross-Lagged Panel Model between Leisure Reading and Parent Reported Prosocial Behavior. *Note.* Numbers represent standardized coefficients with standard errors in parentheses. Coefficients for models that include control variables (migration background, socioeconomic status, nonverbal intelligence) are printed in bold. Coefficients for the reduced sample of students nested in schools are in italics. Model fit: χ^2^ = 368.626, *df* = 86, RMSEA = .025, SRMR = .029, CFI = .995, TLI = .994. Model fit with control variables: χ^2^ = 662.411, *df* = 116, RMSEA = .030, SRMR = .043, CFI = .990, TLI = .988. Nested in schools: Model fit: χ^2^ = 265.228, *df* = 86, RMSEA = .025, SRMR = .035, CFI = .993, TLI = .993. Model fit with control variables: χ^2^ = 410.216, *df* = 116, RMSEA = .027, SRMR = .043, CFI = .989, TLI = .987. * *p* < .05 * *p* < .05 (two-tailed). (**B**) Cross-Lagged Panel Model between Leisure Reading and Parent Reported Peer Problems. Numbers represent standardized coefficients with standard errors in parentheses. Coefficients for models that include control variables (migration background, socioeconomic status, nonverbal intelligence) are printed in bold. Coefficients for the reduced sample of students nested in schools are in italics. Model fit: χ^2^ = 709.730, *df* = 83, RMSEA = .038, SRMR = .041, CFI = .988, TLI = .987. Model fit with control variables: χ^2^ = 1130.740, *df* = 113, RMSEA = .042, SRMR = .065, CFI = .981, TLI = .978. Nested in schools: Model fit: χ^2^ = 404.704, *df* = 83, RMSEA = .034, SRMR = .043, CFI = .988, TLI = .987. Model fit with control variables: χ^2^ = 670.502, *df* = 113, RMSEA = .038, SRMR = .069, CFI = .979, TLI = .975. * *p* < .05 (two-tailed).

Cross-lagged panel models indicated that leisure reading at Grade 6 was again positively correlated to concurrent parent reported peer problems (Fig. 4B). However, we found no significant effect of leisure reading on later parent reported peer problems. In contrast, leisure reading was even—to a small degree—negatively associated to later parent reported prosocial behavior (Fig. 4A). Additional analyses with students nested in schools and including only those students who remained in the same school from Grades 5–9 yielded similar results. An exception was that the association between leisure reading and later prosocial behavior was no longer statistically significant after adding the control variables (see Fig. 4A and B).

### Cumulative leisure reading and parent reported prosocial behavior and peer problems

**Figure 5:**
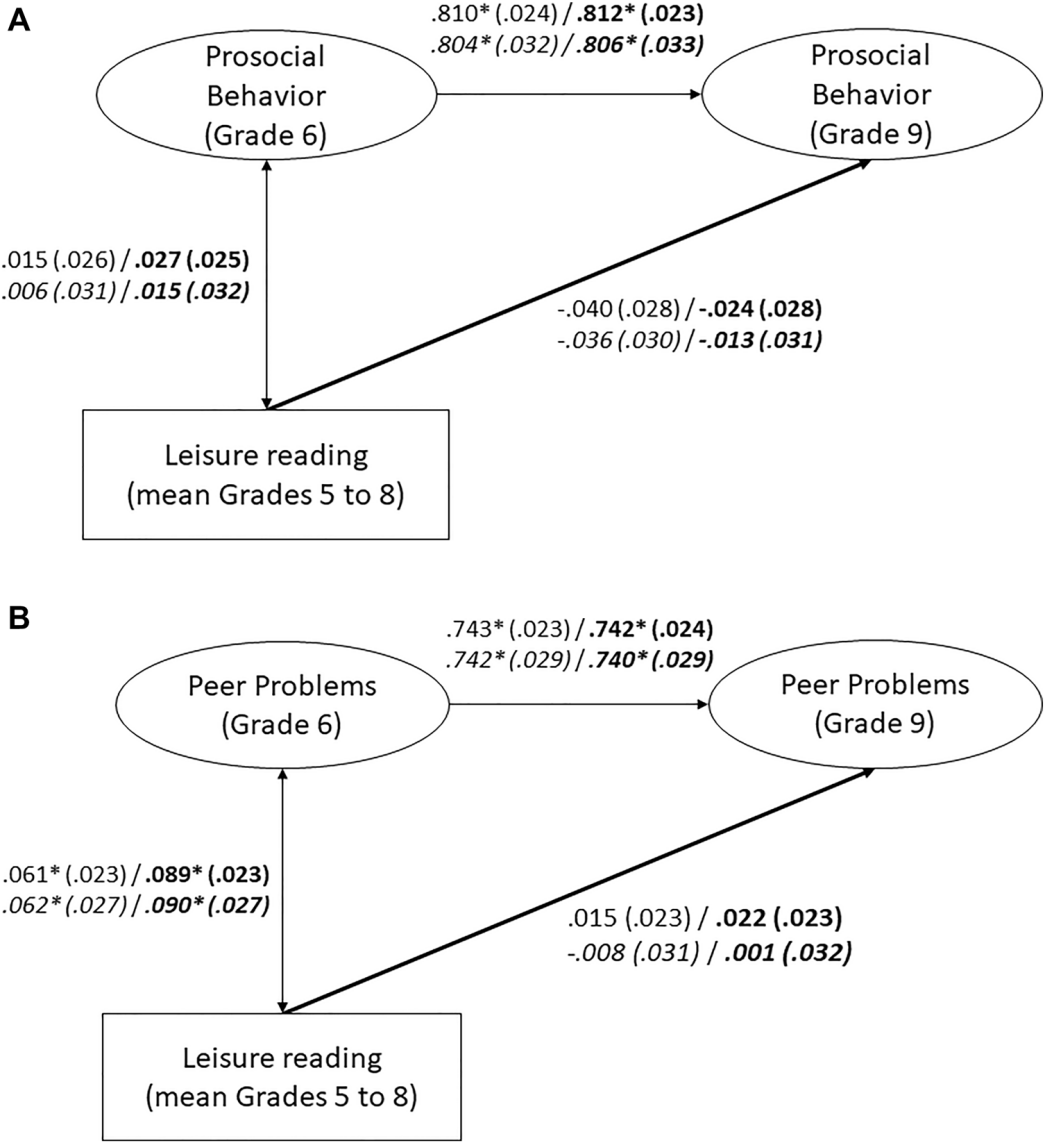
(**A**) Cumulative Leisure Reading and Parent Reported Prosocial Behavior. Numbers represent standardized coefficients with standard errors in parentheses. Coefficients for models that include control variables (migration background, socioeconomic status, nonverbal intelligence) are printed in bold. Coefficients for the reduced sample of students nested in schools are in italics. Model fit: χ^2^ = 248.405, *df* = 50, RMSEA = .028, SRMR = .032, CFI = .980, TLI = .978. Model fit with control variables: χ^2^ = 522.938, *df* = 74, RMSEA = .034, SRMR = .048, CFI = .957, TLI = .949. Nested in schools: Model fit: χ^2^ = 186.061, *df* = 50, RMSEA = .028, SRMR = .037, CFI = .975, TLI = .973. Model fit with control variables: χ^2^ = 309.060, *df* = 74, RMSEA = .031, SRMR = .047, CFI = .958, TLI = .950. * *p* < .05 (two-tailed). (**B**) Cumulative Leisure Reading and Parent Reported Peer Problems. Numbers represent standardized coefficients with standard errors in parentheses. Coefficients for models that include control variables (migration background, socioeconomic status, nonverbal intelligence) are printed in bold. Coefficients for the reduced sample of students nested in schools are in italics. Model fit: χ^2^ = 517.041, *df* = 47, RMSEA = .044, SRMR = .044, CFI = .961, TLI = .954. Model fit with control variables: χ^2^ = 993.913, *df* = 71, RMSEA = .050, SRMR = .076, CFI = .929, TLI = .912. Nested in schools: Model fit: χ^2^ = 309.340, *df* = 47, RMSEA = .041, SRMR = .047, CFI = .961, TLI = .954. Model fit with control variables: χ^2^ = 596.971, *df* = 71, RMSEA = .047, SRMR = .081, CFI = .925, TLI = .907. * *p* < .05 (two-tailed).

Cumulative leisure reading did not significantly predict later parent reported prosocial behavior (Fig. 5A) or peer problems (Fig. 5B). Additional analyses with students nested in schools and including only those students who remained in the same school from Grades 5 to 9 yielded comparable results (see Fig. 5A and B).

### Cumulative leisure reading across different genre and parent reported prosocial behavior and peer problems

**Figure 6:**
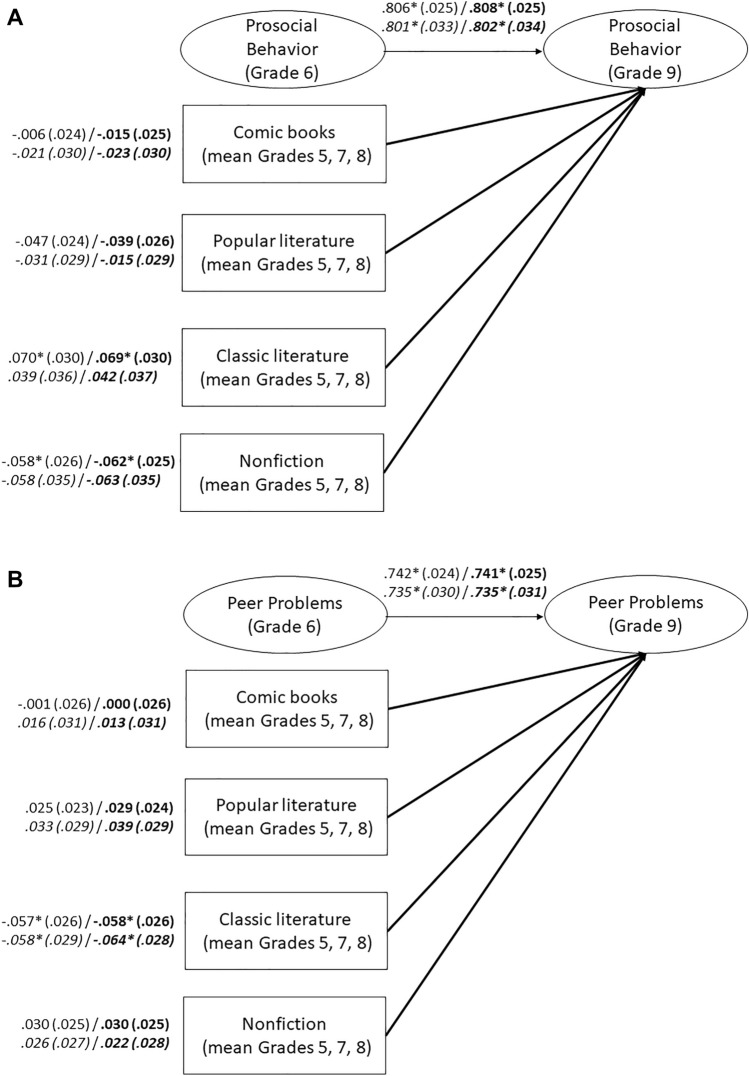
(**A**) Cumulative Leisure Reading across Different Categories and Parent Reported Prosocial Behavior. Numbers represent standardized coefficients with standard errors in parentheses. Coefficients for models that include control variables (migration background, socioeconomic status, nonverbal intelligence) are printed in bold. Coefficients for the reduced sample of students nested in schools are in italics. Model fit: χ^2^ = 460.741, *df* = 74, RMSEA = .032, SRMR = .035, CFI = .966, TLI = .958. Model fit with control variables: χ^2^ = 694.567, *df* = 98, RMSEA = .034, SRMR = .045, CFI = .951, TLI = .933. Nested in schools: Model fit: χ^2^ = 327.137, *df* = 74, RMSEA = .032, SRMR = .040, CFI = .956, TLI = .946. Model fit with control variables: χ^2^ = 438.449, *df* = 98, RMSEA = .032, SRMR = .045, CFI = .941, TLI = .920. * *p* < .05 (two-tailed). (**B**) Cumulative Leisure Reading across Different Categories and Parent Reported Peer Problems. Numbers represent standardized coefficients with standard errors in parentheses. Coefficients for models that include control variables (migration background, socioeconomic status, nonverbal intelligence) are printed in bold. Coefficients for the reduced sample of students nested in schools are in italics. Model fit: χ^2^ = 595.972, *df* = 71, RMSEA = .038, SRMR = .039, CFI = .961, TLI = .951. Model fit with control variables: χ^2^ = 1073.915, *df* = 95, RMSEA = .045, SRMR = .065, CFI = .933, TLI = .906. Nested in schools: Model fit: χ^2^ = 351.424, *df* = 71, RMSEA = .034, SRMR = .042, CFI = .960, TLI = .949. Model fit with control variables: χ^2^ = 631.259, *df* = 95, RMSEA = .041, SRMR = .070, CFI = .926, TLI = .897. * *p* < .05 (two-tailed).

Cumulative reading of nonfiction did not predict later parent reported peer problems (Fig. 6B) and was even negatively related to later parent reported prosocial behavior (Fig. 6A). Consistent with the associations for self-reported prosocial behavior and peer problems, among the narrative genres only cumulative reading of modern classic literature positively predicted greater parent reported prosocial behavior and fewer parent reported peer problems. These statistically significant relations were small in magnitude and the similar predictions we made for popular literature and comic books were not supported. Additional analyses with students nested in schools and including only those students who remained in the same school from Grades 5–9 yielded largely similar results. However, in these analyses none of the reading categories were significantly related to parent reported prosocial behavior (see Fig. 6A and B).

### Replication of all analyses including gender, reading competence, and trait openness to experience as additional control variables

The more conservative methodological approach, in which gender, reading competence, and trait openness to experience were included as additional control variables, replicated the findings of the original analyses, in which only migration background, non-verbal intelligence, and parents’ educational background were included as control variables (see supplemental material on OSF: https://doi.org/10.17605/OSF.IO/D97QN).

## Discussion

We examined whether leisure reading would predict later prosocial behavior and social adjustment in a large, nationally-representative, longitudinal dataset from Germany. Both self-reports (pre-registered) and parental reports (exploratory) were examined. Based on previous theoretical accounts we hypothesized that leisure reading would improve social-cognitive skills and result in positive downstream consequences^8–10^. To examine our hypotheses, we used cross-lagged panel analyses between leisure reading and prosocial behavior/social adjustment from Grades 5 to 9, as well as analyses linking cumulative reading across Grades 5 to 8 to later prosocial behavior/social adjustment in Grade 9. In contrast to our hypotheses, neither leisure reading in Grade 5 nor cumulative leisure reading from Grade 5–8 predicted later prosocial behavior or social adjustment, when controlling for prior prosocial behavior/social adjustment and other important control variables (migration background, education, and nonverbal intelligence). Thus, our results do not align with the assumption that reading frequency in general results in a benefit to social cognition and associated social consequences. However, our study does not represent a strict test of this assumption because these archival data do not include measures of social-cognitive skills (e.g., theory-of-mind or empathy), only putative downstream consequences of these skills. Prosocial behavior and social adjustment represent distal consequences of social cognition that are also likely influenced by many other things (e.g., parenting, socialization, sociocultural factors). In many ways, expecting leisure reading to predict these downstream consequences of social cognition is a rather stiff test. The NEPS data also did not measure the motivation to engage in social processing, and possessing the ability to engage in social cognition is separate from the motivation to do so^41–43^. Even if reading promotes social-cognitive skills, in the absence of the appropriate motivation these downstream consequences would not be observed. Another aspect of our study to note is that many theories propose an effect for reading specific types of text (e.g., fiction or literary fiction in specific), whereas these main analyses examined leisure reading as a whole. That said, our results do partially replicate findings from the UK Millennium Cohort study, in which reading frequency at the age 7 predicted prosocial behavior but not peer problems at age 11^36^. This study also joins a growing body of longitudinal research examining the relation between reading and social outcomes, which has sometimes supported a longitudinal association between the two^44,45^, and sometimes failed to find such an association^46^.

Because the NEPS data also provides information on leisure reading for different genres (nonfiction, popular literature, modern classic literature, comics), we also examined whether genre is relevant for predicting prosocial behavior and social adjustment. Based on the SPaCEN framework, we predicted that all the narrative categories, but not the nonfiction category, would predict these outcomes^8^. Consistent with this prediction, we found that cumulative reading of nonfiction across Grades 5 to 8 did not predict later prosocial behavior or social adjustment. This is consistent with previous cross-sectional correlational research, in which no associations—and sometimes even negative associations—are reported between nonfiction and social-cognitive skills^26,31^. Also as hypothesized, cumulative reading of modern classic literature predicted later prosocial behavior and fewer peer problems. However, this was not observed for the other two narrative categories: popular literature and comic books. Accordingly, our results are a better fit with Kidd and Castano’s notion that only literary fiction, but not popular fiction, improve social-cognitive skills^6,32,37,38^. These authors theorize that literary fiction is unique in its ability to promote social cognition by virtue of “showing” rather than “telling”, and presenting complex three-dimensional characters rather than stereotypical and flat ones. Both techniques should prompt readers to engage in mental inferencing, in order to understand what these types of characters are thinking. Texts that are concrete and explicit, and rely on simple and predictable characters, seem less likely to evoke mental inferences. This finding, that literary fiction is unique in predicting social adjustment and prosocial behavior, would seem to contradict a previous correlational study, which found that reading romance fiction is the most robust predictor of social-cognitive abilities in adults^24^. However, this discrepancy could easily be a function of different populations (i.e., adolescents in these data, rather than adults) and therefore likely different constituting popular fiction^24^. It is also important to note that these effects were small (β ≤ 0.10) and are unlikely to be detected with smaller samples, as we expected in our a-priori power analysis.

As a limitation of the present study, we have to note that school and, in particular, class membership was not stable for a number of students as they progressed from Grade 5 to 9. Accordingly, the pre-registered analytic approach (i.e., students nested in classes and schools) had to be adjusted. We did two complementary analyses, one with the total sample without nesting, and one with a reduced sample with students nested in schools. Both analyses are limited and may result in biased parameter estimates. Importantly, however, both approaches resulted in very similar parameter estimates and led to the same conclusions. This supports the robustness of the observed effects. Cross-classified models are an additional analytic option that might be used to address the problem of non-stable group memberships across time points^47^. However, this would also result in a reduced sample due to missing information on students’ group memberships at several time points. In addition, to our knowledge, *Mplus* cannot yet handle a combination of several cross-classified and nested group memberships, as is required for our data.

Encouragingly, our pattern of findings were largely replicated in our exploratory analyses of the parent reports of prosocial behavior and social adjustment. Moreover, a more conservative analytic approach, controlling for numerous other variables (i.e., gender, students’ reading competence, and trait openness to experience)^24,31^, yielded comparable results for both self-reports and parental reports. This demonstrates that the observed effects, though small in magnitude, are robust.

In sum, our results do not support the assumption that (cumulative) leisure reading in general results in positive downstream consequences associated with better social cognition. However, our results do confirm that reading modern classic literature predicts more prosocial behavior and better social adjustment in the future, with no such beneficial effects observed for nonfiction. This suggest that it is worthwhile to investigate the effects of reading different genres in future research. To do so, a greater number of well-powered longitudinal studies directly examining social-cognitive skills (e.g., theory-of-mind, empathy, emotion recognition, social memory) are clearly needed.

## Methods

### Design and procedure

We used data from the National Educational Panel Study (NEPS): Starting Cohort Grade 5 (10.5157/NEPS:SC3:11.0.1^48^). From 2008 to 2013, NEPS data was collected as part of the Framework Program for the Promotion of Empirical Educational Research funded by the German Federal Ministry of Education and Research (BMBF). As of 2014, NEPS is carried out by the Leibniz Institute for Educational Trajectories (LIfBi) in cooperation with a nationwide network. A detailed description of the project can be found in Blossfeld and colleagues^49^. The NEPS study is conducted under the supervision of the German Federal Commissioner for Data Protection and Freedom of Information and in coordination with the German Standing Conference of the Ministers of Education and Cultural Affairs and—in the case of surveys at schools—the Educational Ministries of the respective Federal States. The data protection unit of the LIfBi checked data collection procedures, instruments, and documents. Participation in the NEPS study was voluntary, and participants could withdraw from the study at any time. All participants gave informed consent.

Our data comes from “Starting Cohort Grade 5”, which provides data starting in Grade 5 (around 10 years of age) and ending in Grade 12. There were nine assessment waves, with each wave being conducted in different school years or grades (Wave 1 in Grade 5, Wave 2 in Grade 6, etc.). As an exception to the rule, two assessment waves were conducted in close succession in Grade 9 (Waves 5 and 6). Quantity of leisure reading was assessed from Waves 1 to 5 (Grades 5 to 9) and reading frequency for specific literary genres was measured in Waves 1 (Grade 5), and Waves 3 (Grade 7) through 5 (Grade 9). Students’ self-reported prosocial behavior and social adjustment were assessed at two measurement points, Wave 2 in Grade 6 and Wave 6 in Grade 9. All of these data are publicly available upon request from the LIfBi.

### Participants

Participants were 6,112 students who were representatively sampled from all children attending the fifth grade in Germany in 2010/2011. A stratified multi-stage sampling based on explicit stratification (schools educating students in Grade 5 and in Grade 9, schools educating students in Grade 5 but not in Grade 9, special-needs schools) and implicit stratification (school type, Federal State, regional classification, funding institution) was employed. Schools were first selected from the strata and then two classes (if available) per school were randomly selected. Data was then collected from entire school classes^50,51^. As students of special-needs schools (570 participants in Grade 5) might differ from other students in a number of ways and were excluded from the panel cohort after Wave 4 (Grade 8), only regular students were included in the present analyses (5,208 participants in Grade 5)^51,52^. Of these students, 48.2% were female (< 1% missing), mean maximum ISCED-97 for parents (a combination of parents’ highest school qualification and their last vocational qualification) across all waves (1 to 6) was 6.46 (25.7% missing), and 2,919 participated in all waves (1 to 6) between Grades 5 and 9^51,53^.

### Measures

#### Demographics

Demographic data were self-reported by students and parents, and included age, gender, migration status, and socioeconomic status. In the current study, migration background was defined as both of the child’s parents being born outside of Germany. Socioeconomic status was based on a combination of parents’ highest school qualification and their last vocational qualification (ISCED-97), ranging on an eleven-point scale from no school-leaving qualification to doctorate or habilitation^54,55^. Socioeconomic status and migration background were used as control variables in the analyses.

#### Leisure reading

The overall frequency of leisure reading was assessed with two self-report items, with one item addressing how much leisure reading takes place on normal school days and the other concerning normal non-school days. Both items were answered on a five-point Likert scale (1 = *not at all* to 5 = *more than 2 h*). Quantity of leisure reading was assessed from Wave 1 to 5 (Grade 5 to 9).

#### Genre

We analysed genre of leisure reading for the following categories: (1) detective novels, thrillers, horror or fantasy (e.g., Harry Potter or Lord of the Rings); (2) modern classic literature (e.g., by George Orwell or Günther Grass); (3) nonfiction; and (4) comic books. One item was employed for each category and responses were made on a five-point Likert scale (1 = *never or seldom* to 5 = *daily*). These data exist for Wave 1 (Grade 5) and Waves 3 (Grade 7) through 5 (Grade 9).

#### Social-cognitive behavior

As indicators of real-life social-cognitive behavior, two subscales from the Strengths and Difficulties Questionnaire (SDQ)^56^ were used: Prosocial Behavior and Peer Problems/Problem Behavior. The former represents prosociality (e.g., “I try to be nice to other people, their feelings are important to me.”; “I am ready to help people when they are injured, sick or sad.”) and the latter social adjustment to the peer group (e.g., “Most of the time I spend alone; I rather concentrate on myself.”; “I have one or several good friends.”). The SDQ is a widely used instrument in research and practical settings, for several disciplines such as medicine, psychology, and education. Its popularity is in part evidenced by the fact it has been translated into several other languages and adapted for different cultures^57,58^. For both measures, we had access to both self- and parental reports that were available at two measurement points, Wave 2 (Grade 6) and Wave 6 (Grade 9). For both measures, we used the self-reports for the pre-registered confirmatory part of the manuscript and the parental reports for additional exploratory analyses. Each subscale consists of five items with responses made using a 3-point Likert scale: *not applicable* (0), *partly applicable* (1), *clearly applicable* (2). For each scale, the sum score ranges between 0 and 10 points. A recent review reports sufficient test–retest reliability for the self-report form of both scales (0.60 and 0.63)^57^. To allow direct comparisons with previous research, we used the self-report version of the SDQ. Another advantage of using the self-report data, instead of the parental report, is that parental reports are unlikely to accurately reflect experiences encountered when students are in school and away from home (unless students tell their parents everything that occurs at school, in relation to prosocial behavior or peer problems). This lack of insight into student experiences while at school may explain why the parental version is only moderately correlated with the self-report version^57^.

#### Non-verbal intelligence

Our control variable of non-verbal intelligence was measured using a matrices test constructed by the NEPS research group (NEPS-MAT), which assesses reasoning^59^. Each of 12 items consists of several horizontally and vertically arranged fields, in which different geometric elements are depicted with one field left empty. The logical rules on which the pattern of shapes is based must be deduced in order to select the right complement for the empty field (akin to Raven’s progressive matrices^60^). The scores range from 0 to 12. The test was administered in Wave 1 (Grade 5) and in Wave 6 (Grade 9). We used the score in Grade 5 as a control variable in the analyses.

### Analyses

All of our analyses were preregistered on AsPredicted (see https://aspredicted.org/8gf5b.pdf) before requesting access to the NEPS data (i.e., we had no access to the data before the registered report had been accepted). We had no knowledge of this dataset other than what is reported in the publicly available documents (e.g., codebook, information on survey instruments; see https://www.neps-data.de/Data-Center/Data-and-Documentation/Start-Cohort-Grade-5/Documentation), which provided no insight into our research questions. The NEPS data are available for public use and can be requested from the Leibniz Institute for Educational Trajectories (LIfBi).

#### Data handling and analysis

#### General procedure for structural equation modeling

The main analyses was conducted with M*plus* (version 8.8)^61^. Our analysis script is publicly available on OSF. As the data are publicly available, all our analyses are replicable in conjunction with this script. Categorical variables were dummy coded. For detecting outliers, we checked for univariate (Median Absolute Deviation > 3) and multivariate outliers (Mahalanobis-Minimum Covariance Determinant with *p* = 0.001) with the R package *Routliers*^62^. As the NEPS data are corrected for implausible values on individual variables and scores, only multivariate outliers (*n* = 56, resulting in a final sample *N* = 5152) were removed before the analyses were conducted. To address non-independence of observations (due to students being nested in classes and schools), we had initially proposed to conduct our analyses with the M*plus* option type set as *complex twolevel*^61^). However, as class attendance varied for many students from Grade 5 to Grade 9, and the school attended also changed for a considerable portion of the sample, we conducted all analyses without nesting. To account for this change in procedure, we additionally performed all analyses with the students who remained in the same school (*N* = 3387, with multivariate outliers removed *N* = 3344), in which we included the nesting in schools (M*plus* option type set as *complex*) The code and results of these additional analyses are also publicly available on OSF. This was a suitable approach because we were interested in relations between variables on student level and did not use any predictors on class or school level. Accordingly, we set the estimator to WLSMV (instead of MLR), which is the default for ordinal variables and which provides information on model fit. Concomitant with the change of the estimator, we used multiple imputation (with covariates included; 40 imputations^63^) to address missing data (instead of FIML, which is used with MLR).

#### Model fit and model comparison

As indicators of model fit, the comparative fit index (CFI), the Tucker–Lewis index (TLI), the root mean square error of approximation (RMSEA), and the standardized root mean square residual (SRMR) were reported. Values of 0.95 or higher for the CFI and TLI, lower than 0.06 for the RMSEA, and lower than 0.08 for the SRMR are indicative of good model fit^64^. We also considered the statistical significance of the Chi-Square test in model evaluation. However, given the large sample size, this test has an extremely high power so that a statistically significant Chi-Square test per se is not indicative of bad model fit. To assess the importance of individual paths, we examined whether the paths were statistically significant and whether model fit decreased when a path was dropped from the model. In addition to an inspection of the model fit indices (CFI, TLI, RMSEA, SRMR), Chi-Square difference tests were calculated to compare nested models. Statistically significant Chi-Square difference tests or CFI, TLI, RMSEA, and SRMR values that no longer indicate good model fit were treated as indicators that model fit had significantly worsened. Further interpretation of the importance of statistically significant individual paths were guided by the size of the path coefficient in the fully standardized solution.

#### Latent variables and measurement invariance testing

The SDQ scales and leisure reading (in the cross-lagged panel models) were modelled as latent variables. For SDQ scales, the five variables were used as indicator variables. For leisure reading, the two items were used as indicator variables. To do so, we tested for longitudinal measurement invariance by comparing a series of nested factor models, adding specific restrictions to the models^65,66^. In a first step, we confirmed configural invariance, which indicates an equivalent factor structure across time. In a second step, we tested for weak factorial invariance, constraining the factor loading over time to be equal. In a third step, we tested for strong factorial invariance, also constraining the thresholds over time to be equal. In a final step, strict invariance was tested, establishing that the residual variances were also equivalent across time. Changes in CFI greater than − 0.01 and in RMSEA greater than 0.015 were considered a meaningful decrease in model fit^67^. If weak invariance could be established, analyses were conducted with latent variables. In addition, in a next step, the variables were examined for partial measurement invariance^65,68^. Finally, If weak invariance could not have been established, we had planned to use a parceling approach (i.e., the *balancing approach*^69^), but that was not necessary. Parceling has several advantages over alternative methods, such as higher reliability, lower likelihood of distributional violations, and fewer parameter estimates^69^.

#### Hypothesis testing

#### Examining leisure reading: cross-lagged panel design analyses

To test the hypotheses that leisure reading predicts future prosocial behavior/social adjustment while controlling for earlier prosocial behavior/social adjustment and other control variables (non-verbal intelligence, migration background, socioeconomic status), we modelled the relationship between leisure reading and prosocial behavior/social adjustment in a two-way cross-lagged panel design (see Fig. 1). The analyses were conducted separately for prosocial behavior and social adjustment. Prosocial behavior/social adjustment and leisure reading were treated as latent variables.

#### Does leisure reading predict future prosocial behavior?

We conducted two-way cross-lagged panel design analyses that examined associations between leisure reading (Grade 6 and 9) and prosocial behavior (self-reports; Grade 6 and 9), controlling for non-verbal intelligence, migration background, and socioeconomic status.

#### Does leisure reading predict future social adjustment?

We conducted two-way cross-lagged panel design analyses (analogous to the analysis of prosocial behavior) that examined associations between leisure reading (Grade 6 and 9) and social adjustment (self-reports; Grade 6 and 9), controlling for non-verbal intelligence, migration background, and socioeconomic status.

#### Examining leisure reading: cumulative analyses

To test the hypotheses that cumulative leisure reading predicts future prosocial behavior/social adjustment while controlling for earlier prosocial behavior/social adjustment and other control variables (non-verbal intelligence, migration background, socioeconomic status), we examined cumulative leisure reading across several grades using SEM (see Fig. 2). The analyses were conducted separately for prosocial behavior and social adjustment. The SDQ scales were treated as latent variables. Cumulative leisure reading was modelled as a manifest variable, averaging all items across Grades 5 to 8 (in analogy to the treatment of cumulative practice in expertise research^70^).

#### Does cumulative reading predict future prosocial behavior?

We constructed structural equation models examining associations between leisure reading (mean of Grades 5 to 8) and prosocial behavior (self-reports; Grade 9), controlling for earlier prosocial behavior (self-report; Grade 6), non-verbal intelligence, migration background, and socioeconomic status.

#### Does cumulative reading predict future social adjustment?

We constructed structural equation models that examined associations between leisure reading (mean of Grades 5 to 8) and social adjustment (self-reports; Grade 9), controlling for earlier social adjustment (self-report; Grade 6), non-verbal intelligence, migration background, and socioeconomic status.

#### Examining the role of genre: cumulative analyses

We examined whether there were differences between genres in effects on prosocial behavior and social adjustment, focusing on classic literature, popular literature, comic books, and nonfiction (see Fig. 3). To do so, we conducted two separate structural equation models: one for prosocial behavior and one for social adjustment. Prosocial behavior/social adjustment (self-reports; Grade 9), was predicted by cumulative reading experience in each of the four genre categories (averaged across Grades 5, 7, and 8), controlling for previous social-cognitive behavior (respective SDQ scales; measured in Grade 6) and other control variables (non-verbal intelligence, migration background, socioeconomic status). Reading genres was treated as manifest variables and averaged across grades. SDQ scales was treated as latent variables.

#### Does genre differentially relate to future prosocial behavior?

We constructed structural equation models examining associations between leisure reading of different genre (mean of Grades 5, 7, and 8) and prosocial behavior (self-reports; Grade 9), controlling for earlier prosocial behavior (self-report; Grade 6), non-verbal intelligence, migration background, and socioeconomic status.

#### Does genre differentially relate to future social adjustment?

We constructed structural equation models examining associations between leisure reading of different genre (mean of Grades 5, 7, and 8) and social adjustment (self-reports; Grade 9), controlling for earlier prosocial behavior (self-report; Grade 6), non-verbal intelligence, migration background, and socioeconomic status.

#### Power analysis

Due to the archival nature of these data an a priori power analysis before data collection was not possible. We performed a power analysis for each type of SEM with the R package *simsem* (version 0.5–16), which provides a framework for Monte Carlo simulations of structural equation models (script is available on OSF)^71^. As we had little information on the cluster structure of the NEPS data, no knowledge about expected ICCs at level 2 (class) and 3 (school), we used a simulation without clustering. Using the *lavaan* option in *simsem*, we first specified a population model for each of the planned analyses (with and without covariates, separately) that was informed by the previous literature^2,11,24,25,35,72–77^. Then we specified corresponding analysis models. Given a power of 0.95, a significance threshold of 0.05, and a standardized path coefficient of 0.10 between reading and social-cognitive behavior^11,25,26,31^, around 2,350 participants were needed for detecting the path of interest in the cross-lagged model (with and without covariates). Around 2,100 participants were needed for the path of interest in the cumulative model (with and without covariates). Finally, in the cumulative genre model, around 3,900 participants were needed for any of the individual paths of interest (with and without covariates). As suggested by the editorial office, we additionally performed a power analysis with a power of 0.80 for each type of SEM, which is a conventionally used threshold for power calculations in psychological research^78^. Given a power of 0.80, around 1450 participants were needed for the cross-lagged model, around 1300 for the cumulative model, and 2350 for the cumulative genre model.

### Exploratory analyses

In exploratory analyses, we repeated all aforementioned analyses using the parental reports of the SDQ scales instead of the self-reports, which were used for confirmatory hypothesis testing. In addition, we explored the robustness of our findings, by including gender, reading competence, and trait openness to experience as additional control variables in the analyses.

#### Openness to experience

Our control variable of openness to experience (which was included in additional exploratory analyses to explore the robustness of our findings) was assessed with two self-report items: “I do not care much about arts” and “I have an active imagination, I am an imaginative person.” Both items were taken from the Big Five Inventory-10 (BFI-10)^79^ and were answered on a five-point Likert scale (1 = *does not apply at all* to 5 = *applies completely*). The first item was reversed, and then both averaged to obtain a single score, ranging from 1 to 5. Openness to experience was assessed via self-reports at Wave 3 and 5, and parental reports at Wave 3 and 6. The Openness to Experience subscale of the German BFI-10 showed adequate test–retest reliability (*r*_tt_ = 0.78) and a high correlation with the standard 9-item scale from the BFI-44 (*r* = 0.80)^79^. We used the self-report score of the earliest assessment (in Wave 3) as a control variable in the analyses.

#### Reading competence

Our control variable of reading competence (which was included in additional exploratory analyses to explore the robustness of our findings) was measured in this dataset using a task constructed by the NEPS research group. It consists of five different text types: informational, commenting or argumenting, literary, instructional, and advertising. Test items for each kind of text rely on different cognitive processes (e.g., finding information, drawing conclusions)^80^. Three different item formats were employed: (1) multiple-choice items; (2) true or false statements; and (3) matching tasks, where a partial title must be assigned to the appropriate section of a text (as an example). A partial credit model was used for scaling the data and manifest scale scores are provided in the form of weighted likelihood estimates^81^. Reading competence was assessed at Wave 1 (Grade 5), Wave 3 (Grade 7), Wave 6 (Grade 9) and Wave 9 (Grade 12). The reading competence tests exhibit high reliability (WLE reliability = 0.77 to 0.79 per wave)^81–83^. We used the score in Grade 5 as control variable in the analyses.

## Data Availability

The NEPS data are publicly available upon request from the LIfBi. https://www.neps-data.de/Data-Center/Data-Access.

## Code availability

Our analysis scripts are made publicly available on OSF: https://doi.org/10.17605/OSF.IO/D97QN.
